# A Novel Roll and Pitch Estimation Approach for a Ground Vehicle Stability Improvement Using a Low Cost IMU

**DOI:** 10.3390/s20020340

**Published:** 2020-01-07

**Authors:** Malik Kamal Mazhar, Muhammad Jawad Khan, Aamer Iqbal Bhatti, Noman Naseer

**Affiliations:** 1 School of Mechanical & Manufacturing Engineering (SMME), National University of Sciences & Technology (NUST), Islamabad 44000, Pakistan; jawad.khan@smme.nust.edu.pk; 2 Department of Electrical Engineering, Capital University of Science & Technology (CUST), Islamabad 44000, Pakistan; aib@cust.edu.pk; 3 Department of Mechatronics, Air University (AU), Islamabad 44000, Pakistan; Noman.naseer@mail.au.edu.pk

**Keywords:** attitude estimation, observer, vehicle dynamics, state estimation, roll angle

## Abstract

Onboard attitude estimation for a ground vehicle is persuaded by its application in active anti-roll bar design. Conventionally, the attitude estimation problem for a ground vehicle is a complex one, and computationally, its solution is very intensive. Lateral load transfer is an important parameter which should be taken in account for all roll stability control systems. This parameter is directly related to vehicle roll angle, which can be measured using devices such as dual antenna global positioning system (GPS) which is a costly technique, and this led to the current work in which we developed a simple and robust attitude estimation technique that is tested on a ground vehicle for roll mitigation. In the first phase Luenberger and Sliding mode observer is implemented using simplest roll dynamics model to measure the roll angle of a vehicle and the validation of results is carried using commercial software, CarSim^®^ (CarSim, Ann Arbor, MI, USA). In the second phase of research, complementary and Kalman filters have been designed for attitude estimation. In the third phase, a low-cost inertial measurement unit (IMU) is mounted on a vehicle, and both the complementary filter (CF) and Kalman filter (KF) are applied independently to measure the data for both smooth and uneven terrains at four different frequencies. We compared the simulated and real-time results of roll and pitch angles obtained using the complementary and Kalman filters. Using the proposed method, the achieved root mean square error (RMSE) is less than 0.73 degree for pitch and 0.68 degree for roll, with a sample time of 2 ms. Thus, a warning signal can be generated to mitigate roll over. Hence, we claim that our proposed method can provide a low-cost solution to the roll-over problem for a road vehicle.

## 1. Introduction

Nowadays, one major objective in road transport systems is to reduce the number of accident victims. For the same reason, vehicles on the market are equipped with control system variants, such as ESC (Electronic Stability Control) and RSC (Roll Stability Control) [1,2] for the improvement of vehicle safety standards. The discussed systems must have knowledge of expected vehicle behavior in advance during different conditions and maneuvers for the proper actuation of the control system [3,4,5]. Specifically, knowledge about the roll angle of the vehicle is useful in RSC systems. Rollover accidents are responsible for nearly 33% of all deaths from passenger vehicle crashes [6].

The RSC system success depends on the vehicle roll angle knowledge. Dual-antenna GPS can be used to directly measure vehicle roll angle, however, the equipment cost for such design is high [7]. For this reason, roll angle needs to be estimated with a cost-effective system [8,9]. For this, there are three approaches [6,10] that can be used to estimate attitude of a vehicle: (1) indirect approach; (2) vehicle model approach; (3) additional sensor aided approach. The indirect approach uses vehicle sensors such as wheel speed sensors and inertial measurement unit (IMU) [9]. The in-vehicle sensor approach is the cheapest solution to vehicle attitude estimation problem but suffers from cumulative integration errors due to sensor bias. The vehicle model approach [11] requires the accurate vehicle model as well as parameters in addition to bias compensation for precise estimation. The additional sensor-aided approach [12] such as using a vision sensor can provide the heading angle of the vehicle. However, the update rate of the vision sensor is too slow, and the failure of camera can frequently occur due to the influence of road conditions as well as weather change.

In [13], an algorithm to estimate the vehicle roll angle is proposed which uses the measurements obtained from suspension deflection sensors and accelerometers. However, this is not a precise estimation method. Furthermore, suspension deflection sensors present on the market are quite expensive and are typically not available for standard vehicles. In [10], a dynamic observer is proposed which utilizes the information obtained through a lateral accelerometer and a gyroscope. However, error is found in the transient response of estimated vehicle roll angle. In the mentioned algorithm, neither model nor measurement noise is taken into account. An observer-based estimator that estimates the accelerometer biases online is proposed in [14]. An estimation method for lateral velocity and attitude has been proposed for automated driving vehicle in [15]. This method fuses the information from the six DOF integrated IMU and vehicle dynamics, and it can run autonomously without aid from extra information. The root mean square error (RMSE) for roll angle in this technique remained at 0.6 degree. Several non-linear attitude estimation techniques such as extended Kalman Filter, multiplicative Kalman Filter, backward smoothing extended Kalman filter, QUEST and recursive QUEST were discussed by [16] showing discrepancies in using each individual algorithm based on computational burden, convergence time and noise response. Filters such as extended Kalman fail where priori estimate is not accurate or highly nonlinear dynamics intervene. Similarly, unscented filters are attractive for nonlinear dynamics without accurate priori estimates but lag in terms of computational burden. Particle filters face dimensionality constraint if more than few parameters are estimated. Such approaches tend to be promising in complex systems where higher computational power along with accurate priori estimates are available. Hence, simpler models-based observers and attitude estimation algorithm with low computational power and less convergence time is proposed in this research.

Two requirements must be met for the design of sensor for driving applications. First, the sensor should be capable enough provide continuous measurement under all circumstances. For ground vehicle applications, it is not feasible to use Global Positioning System (GPS) due to outage of signal in urban region, tunnels and Parking lots etc. [17,18]. Secondly, the sensors must show robust performance under all vehicle maneuvers regardless of their duration or severity. Consequently, low cost inertial navigation systems (INS) because their estimation error tends to grow under prolonged and severe maneuvers [19].

Gyroscopes graded for automotive applications are the simplest and most precise sensors to determine attitude of vehicle. A tri-axial gyroscope can be used to measure angular velocities along all three axes which are integrated with respect to time to obtain the attitude angles. This integration process is used to accumulate any noise or bias, if present in the sensor measurements that can drift away with time. Hence, a near perfect measurement requirement is imposed on the gyroscope. Automotive gyroscopes use fiber-optic and ring laser technology to deliver the accuracy level necessary for driving applications. However, they are extremely expensive and sometimes their cost is comparable to the price of the vehicle itself.

In this paper, we addressed the main issues about state estimation of vehicle roll angle. Vehicle attitude estimation is challenging due to rough terrain, road disturbances and noise. Model based observers are often too complicated to implement and the computation cost of such systems requires a powerful onboard processor. We simulated the vehicle dynamics and narrowed down the complex model to simple one using which the use of observers is computationally less intensive.

The methods proposed are fast and easy to implement, since they are free from iteration. In order to decrease significant effects of gyro bias, we suggested a bias compensation which is merged with filtering architecture. We validated the experiments carried out on real hardware in real time environment to show the performance comparison. Results show that the proposed Filters can estimate with as much accuracy as Kalman filter and higher order observers. We successfully tested the proposed algorithms in ordinary road conditions where we find a balance between estimation accuracy and time consumption. Compared with previously used iterative methods, the proposed filter has much less convergence time.

The introduction of MEMS (micro-electro-mechanical systems) technology has helped in shrinking the size and cost of inertial sensors [20]. Now miniature accelerometers and gyroscopes are available at a very low cost, but their performance is too much degraded for the use in automotive applications. While the drift performance of an automotive grade gyroscope is typically 1°/h, a MEMS gyroscope provides a typical performance of 70°/h [21]. Moreover, this bias is subject to temperature variations as well [22]. Although much work is being done in order to improve the MEMS gyroscope quality, and to make them robust to different vibrations and noise sources for automotive applications [23], the drift issue remains a major concern. A comparison of the performance characteristics for automotive grade MEMS gyroscopes is presented in Table 1 [24].

The trend of performance deterioration resulting from the decrease in cost is easily noticeable considering the TG6000 as most expensive and MPU-6050 as least. Even a nominal automotive grade gyroscope costs approximately 10,000 USD. Several sensors such as camera [25], LIDAR [26], and GPS [27] have been extensively used for attitude estimation for both static and accelerated vehicles. However, these are costly and involve intensive computational algorithms. 

The goal of this paper is to enable the low cost gyroscope (price in the range of 10 USD) to function precisely in ground vehicle related applications. Results obtained using this research are compared to previously documented studies shown in the discussion section, based on which we concluded that our proposed method has a more significant outcome.

## 2. Materials and Methods

**Figure 1:**
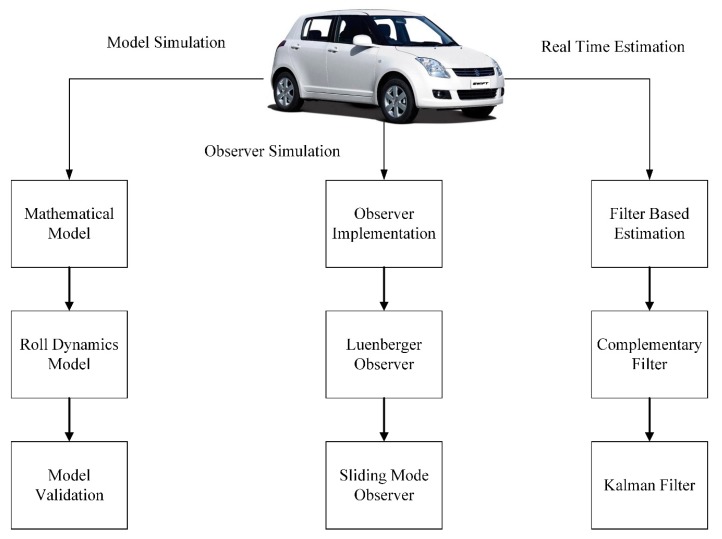
Overall System Architecture.

The InvenSense MPU-6050 (TDK InvenSense, San Jose, CA, USA) is a low-cost sensor which contains a MEMS gyroscope and a MEMS accelerometer mounted on a single chip. It uses the standard I2C-bus Interface. Processing hardware is attached to MPU-6050 and interfaced with the MATLAB® (The MathWorks, Inc., Torrance, CA, USA) using the instrument and control toolbox. The sensor is placed at approximate center of gravity location of the vehicle and the results are acquired under different urban road conditions. Figure 1 shows the overall system architecture and the breakup of research carried out. A mathematical model is developed using dynamics equations to show model consistency. The observer-based approach focuses on estimation carried using plant model (i.e., vehicle model) while real time estimation used sensor with on board motion processor to validate the results obtained by applying filters.

### 2.1. Mathematical Modelling and Validation

#### Mathematical Modeling

**Figure 2:**
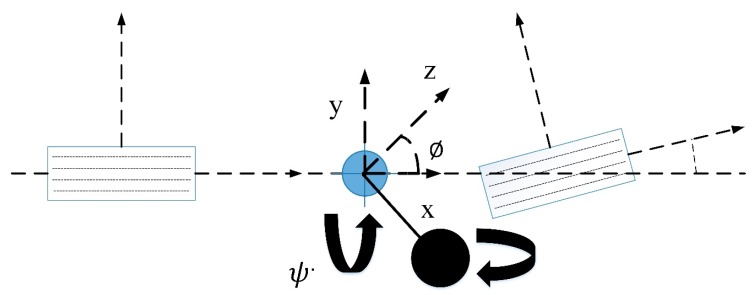
Track Vehicle Model.

For implementation of our algorithm, we used a simple bicycle model with roll dynamics (see Figure 2). Considering roll dynamics, equation for lateral motion is:

(1) $${\mathrm{ma}}_{y}={\mathrm{Fy}}_{f}+{\mathrm{Fy}}_{r},$$

where m is the mass of vehicle. $a_{y}$ is the lateral acceleration, ${\mathrm{Fy}}_{f}$ is front tire force and ${\mathrm{Fy}}_{r}$ is rear tire force. yaw rate becomes:

(2) $$I_{z}\ddot{\psi }=l_{f}{\mathrm{Fy}}_{f}-l_{r}{\mathrm{Fy}}_{r,}$$

where $I_{z}$ is the yaw inertia, $\ddot{\psi }$ is the yaw rate, $l_{f}$ is distance of front from center of gravity and $l_{r}$ is the distance of rear from center of gravity of vehicle. roll rate can be written as,

(3) $$I_{\mathrm{xx}}\dot{\phi }+C_{q}\phi^{.}+K_{q}\phi ={\mathrm{mha}}_{y}$$

where, $I_{\mathrm{xx}}$ is roll moment of inertia, $\dot{\phi }$ is the roll rate, and ϕ is the roll angles, m is the mass of vehicle, and h is the height from center of gravity. $C_{q}$ is the compliance and $K_{q}$ is the stiffness coefficient.

Substituting the value of $a_{y}$, ${\mathrm{Fy}}_{f}$, and ${\mathrm{Fy}}_{r}$, the above equations for lateral acceleration becomes:

(4) $$\dot{v_{y}}=\frac{C_{f}}{m\left[\delta -\frac{v_{y}+\mathrm{Lf}\left(\dot{\psi }\right)}{v_{x}}\right]}+\frac{C_{r}}{m\left[-\frac{v_{y}-\mathrm{Lr}\left(\dot{\psi }\right)}{v_{x}}\right]}$$

where $\dot{v_{y}}$ is the lateral acceleration δ is the steer angle $\dot{\psi }$ is the yaw rate, $v_{x}$ is the longitudinal velocity, and $v_{y}$ is the lateral velocity.

Similarly, yaw acceleration can be expressed as:

(5) $$\ddot{\psi }=\frac{{\mathrm{aC}}_{f}}{I_{\mathrm{zz}}\left[\delta -\frac{v_{y}+\mathrm{Lf}\left(\dot{\psi }\right)}{v_{x}}\right]}-\frac{{\mathrm{bC}}_{r}}{I_{\mathrm{zz}}\left[-\frac{v_{y}-\mathrm{Lr}\left(\dot{\psi }\right)}{v_{x}}\right]}$$

where $\ddot{\psi }$ is the acceleration $I_{\mathrm{zz}}$ is the yaw moment of inertia, $v_{x}$ is the longitudinal velocity and $v_{y}$ is the lateral velocity.

Roll Acceleration for a single-track model can be expressed as,

(6) $$\ddot{\phi }=\frac{\mathrm{mh}}{I_{\mathrm{xx}}\left[v_{y^{.}}+v_{x}\psi^{.}\right]}-\frac{C_{q}\phi^{.}}{I_{\mathrm{xx}}}-\frac{K_{q}\phi }{I_{\mathrm{xx}}}$$

where $\ddot{\phi }$ is the roll acceleration, $I_{\mathrm{xx}}$ is the roll moment of inertia.

### 2.2. Observer Based Estimation

State observer provides estimate of internal state of the system. Sensors normally measures the state of the system which may contain noise. An observer is used to obtain a filtered version of state by using actual measurements as well as estimated measurements from output.

#### 2.2.1. Luenberger Observers

Luenberger structure [28] is used for parameter estimation using following equations.

Continuous Time System:

The state space form for observer design for a vehicle is given as:

(7) $$\dot{x}=\mathrm{Ax}+\mathrm{Bu}$$

(8) y=Cx

where the matrices A, B, and C are state space model parameters. The Luenberger observer for such system can be given as,

(9) $$x^{.}=A\hat{x}+\mathrm{Bu}+L\left(y-C\hat{x}\right)$$

Estimation error can be expressed as,

(10) $$e=x-\hat{x}$$

**Figure 3:**
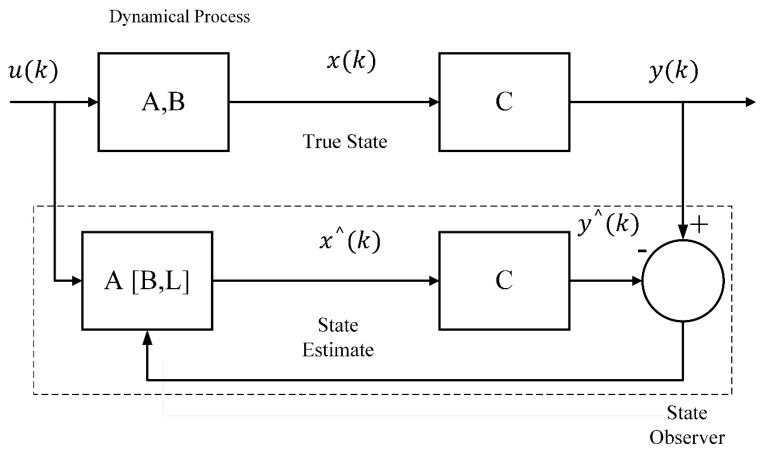
Block Diagram of Observer Implementation.

The Luenberger observer implementation can be seen as a block diagram in Figure 3.

Roll rate error dynamics can be expressed as,

(11) $$e^{.}=\left(A-\mathrm{LC}\right)e=\hat{A}e.$$

**Figure 4:**
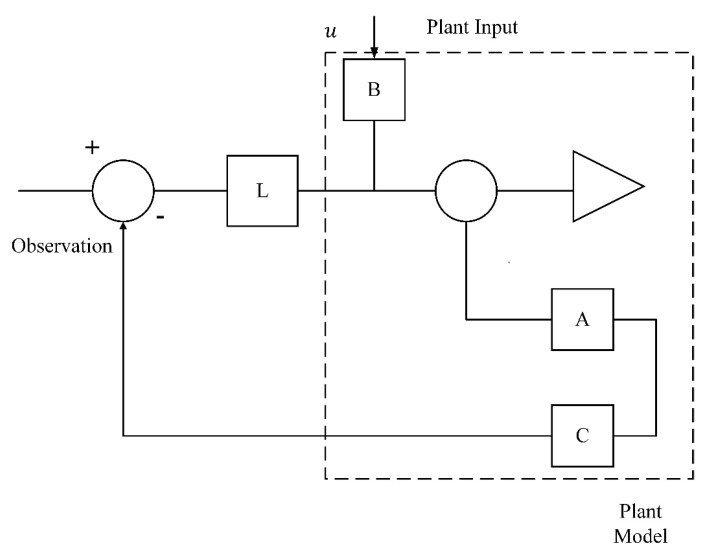
Order Observer.

The estimation error will only converge to zero if $\hat{A}=\left(A-\mathrm{LC}\right)$ has the eigen values which fall in the left plane. L is observer gain matrix and can be obtained using pole placement method. The full order observer block implementation can be seen in Figure 4.

Single-track model roll acceleration can be written as,

(12) $$\phi^{..}=\frac{\mathrm{mh}}{I_{\mathrm{xx}}\left[v_{y^{.}}+v_{x}\psi^{.}\right]}-\frac{C_{q}\phi^{.}}{I_{\mathrm{xx}}}-\frac{K_{q}\phi }{I_{\mathrm{xx}}}.$$

Assuming $a_{y}=v_{y^{.}}+v_{x}\psi^{.}$,

The state space equation form can be written as follows. Let,

(13) $$x_{1}=\phi$$

After differentiation the above equation becomes,

(14) $$x_{1}^{.}=\phi^{.}=x_{2}.$$

Differentiating again the equation can be written as,

(15) $$x_{1}^{..}=\phi^{..}=x_{2}^{.}$$

(16) $$\left[\begin{array}{c} x_{1}^{.}\\ x_{2}^{.} \end{array}\right]=\left[\begin{array}{cc} 0 & 1\\ -\frac{K_{q}}{I_{\mathrm{xx}}} & -\frac{C_{q}}{I_{\mathrm{xx}}} \end{array}\right]\left[\begin{array}{c} x_{1}\\ x_{2} \end{array}\right]+\left[\begin{array}{c} 0\\ \frac{\mathrm{mh}}{I_{\mathrm{xx}}} \end{array}\right]a_{y}$$

where $a_{y}$ is the system input, $I_{\mathrm{xx}}$ is the Roll Inertia. Parameter $K_{q}$ is the spring constant and $C_{q}$ is the damping constant.

The output equation can be written as,

(17) $$y=\left[0\;1\right]\left[\begin{array}{c} x_{1}\\ x_{2} \end{array}\right]$$

Observer Design

Step 1:

After expressing the system in state space form, system observability is investigated. Observability is the property by which the state variables can be estimated from the knowledge of input u(t) and output y(t).

For any n×n matrix A, and for any p×n matrix C observability matrix for any state space system can be expressed as,

(18) $$O=\left[\begin{array}{c} C\\ \mathrm{CA}\\ \begin{array}{c} {\mathrm{CA}}^{2}\\ .\\ \begin{array}{c} .\\ {\mathrm{CA}}^{n-1} \end{array} \end{array} \end{array}\right]$$

If matrix rank of O is equal to that of n than we can say that system under consideration is observable.

Step 2:

For the convergence of estimation error to zero, $\hat{A}=A-\mathrm{LC}$ must have all its eigen values in the left-half plane. Based on this, the poles or eigen values of the system matrix A are computed.

Step 3:

To start with observer design first, we must select the gain matrix L. We will use the pole placement method for this purpose. Matlab^®^ (The MathWorks, Inc., Torrance, CA, USA) command L = place (A’, C’, [p])’ places the desired closed loop poles p by computation of a state feedback gain matrix L.

Step 4:

$\hat{A}=\left(A-\mathrm{LC}\right)$ obtained is our new system matrix, where L in our case is a 2×1 matrix.

$B_{\mathrm{new}}=\left[B\;L\right]\left[\begin{array}{c} u\\ y_{e} \end{array}\right]$, where u is referred as the system input and $y_{e}=y-C\hat{x}$.

#### 2.2.2. Sliding Mode Observer

Luenberger observer is precise for the linear systems with low noise. As soon as noise increased the system starts losing track of desired output. In order to resolve this issue, we need observer with noise rejection property that can deal with complex systems as well. Hence, we opted for the sliding mode observer, which is more robust in terms of performance. Experiments performed concluded that sliding mode observer (SMO) kept better system track in all scenarios.

Sliding mode observer [29] was used to estimate the roll angle for ground vehicle. Consider a continuous time system,

The state space can be written as,

(19) $$\dot{x}=\mathrm{Ax}+\mathrm{Bu}$$

(20) y=Cx

(21) $$T_{c}=\left[\begin{array}{c} N_{c}^{T}\\ C \end{array}\right]$$

where the columns of $N_{c}\in {\mathbb{R}}^{n\times \left(n-p\right)}$ span the null space of C. 

The non-singular transformation, ${\mathrm{CT}}_{c}^{-1}=\left[\begin{array}{cc} 0 & I_{p} \end{array}\right]$ becomes the new distribution matrix. The other system matrices can be expressed as, $T_{c}{\mathrm{AT}}_{c}^{-1}=\left[\begin{array}{cc} A_{11} & A_{12}\\ A_{21} & A_{22} \end{array}\right]$ and $T_{c}B=\left[\begin{array}{c} B_{2}\\ B_{2} \end{array}\right]$ where $T_{c}x=\left[\begin{array}{c} x\\ y \end{array}\right]$.

Then, the nominal system can be written as under.

(22) $$\dot{x}=A_{11}x+A_{12}y+B_{1}u$$

(23) $$\dot{y}=A_{21}x+A_{22}y+B_{2}u$$

Considering the state matrix represented in Equation (16),

$$\left[\begin{array}{cc} A_{11} & A_{12}\\ A_{21} & A_{22} \end{array}\right]=\left[\begin{array}{cc} 0 & 1\\ -\frac{K_{q}}{I_{\mathrm{xx}}} & -\frac{C_{q}}{I_{\mathrm{xx}}} \end{array}\right]$$

Similarly,

$$\left[\begin{array}{c} B_{1}\\ B_{2} \end{array}\right]=\left[\begin{array}{c} 0\\ \frac{\mathrm{mh}}{I_{\mathrm{xx}}} \end{array}\right]$$

The proposed Utkin observer has the form,

(24) $$\dot{\hat{x}}=A_{11}\hat{x}+A_{12}\hat{y}+B_{1}u+\mathrm{Lv}$$

(25) $$\dot{\hat{y}}=A_{21}\hat{x}+A_{22}\hat{y}+B_{2}u-v$$

Vector L is computed in such a way that $A_{11}+{\mathrm{LA}}_{21}$ lies in spectrum C. The discontinuous vector v is defined by,

(26) $$v=M\;\mathrm{sgn}\left(\hat{y}-y\right)$$

**Figure 5:**
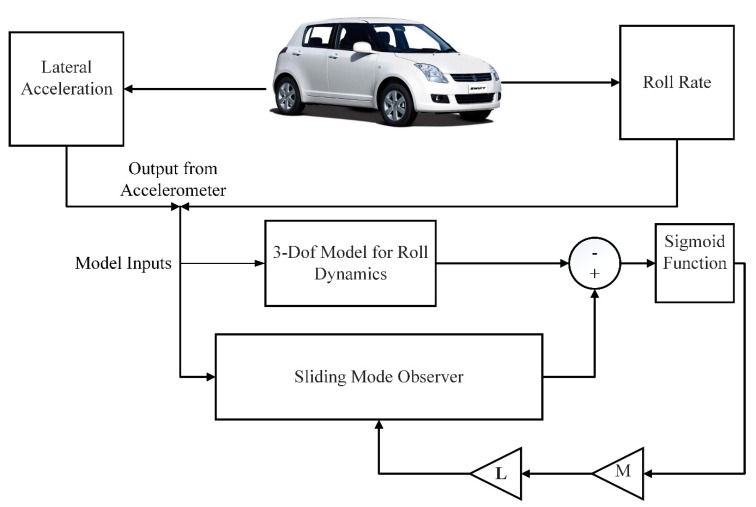
Sliding Mode Observer Estimation Process.

The value of variable M used in experiments remained 0.1. The overall block diagram for sliding mode observer can be seen in Figure 5.

### 2.3. Filter Based Estimation

#### 2.3.1. The Complementary Filter

In order to get rid of complex vehicle models and observers as well as state estimators, we opted for a simple, yet precise estimation technique known as complementary filters. Complementary filter is a fusion algorithm which provides best among the measurements [30]. The gyroscope data will be used during short term maneuvers because of its precision and such data obtained is not liable to be influenced by external forces. Whereas during the long-term maneuvers, we will rely on the data obtained from the accelerometer as it does not drift. Equation (27) provides the simplest form for complementary filters

(27) angle=α∗(angle+gyroData∗dt)+(1−α)∗accData)

**Figure 6:**
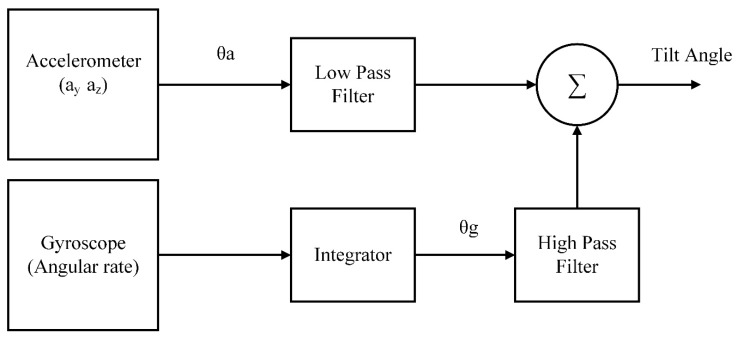
Complementary Filter Configuration.

The overall block diagram for complementary filter can be seen in Figure 6 for better understanding. Based on the value of current angle, the gyro data will be integrated over every time step after this process it will be fused with the low pass filter data relying on accelerometer. The constants α and 1−α are tuning parameters of filter and are chosen such a way that their sum would add up to 1.

The implementation of complementary filter is carried out using a MPU6050 Inertial measurement unit (IMU) in real time environment. After every iteration the roll and pitch angle values are updated based on the gyroscope values that are obtained by integration with time. Filter update is performed for roll and pitch angles of the gyro data by assigning a percentage weight for the current value with the addition to percentage weight of the angle obtained by the accelerometer to ensure that the measured angle will not drift, keeping it very precise for short- and long-term maneuvers.

Second-Order Complementary Filter:

The roll angle estimate is obtained by blending two preliminary estimates which are each valid in different operation range. In this blending process the weights are selected in such a manner that it always favors the estimate which is closely precise. Roll angle estimate can be obtained by the integration of the measured and compensated roll rate Equation (33). From Equation (37), we get a simplified equation for roll angle estimate by making the assumption that $v_{y}$ is small. The following kinematic relation can be obtained.

(28) $$\emptyset_{\mathrm{st}}=\sin^{-1}\left(\frac{\omega_{z}v_{x}-a_{y}}{g}\right)$$

**Figure 7:**
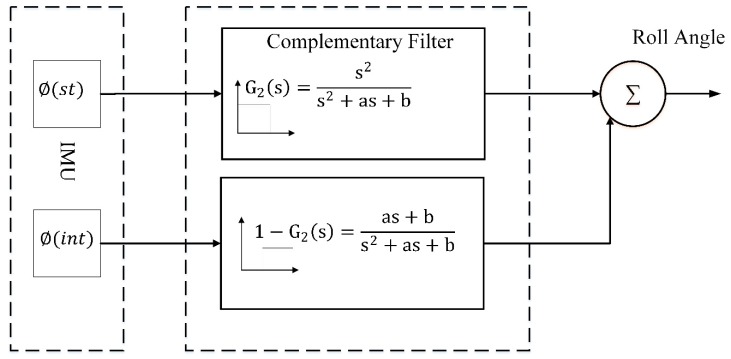
Estimation Process from Gyro and Static Equation.

The second-order complementary filter block diagram can be seen in Figure 7 for real time implementation. Since both the filters should add up to have a unity gain, the low pass filter for such system can be written as,

(29) $$G_{2}\left(s\right)=\frac{s^{2}}{s^{2}+\mathrm{as}+b}$$

where the high pass filter is expressed as

(30) $$1-G_{2}\left(s\right)=\frac{\mathrm{as}+b}{s^{2}+\mathrm{as}+b}$$

The complementary filtering technique discussed in the manuscript uses manually fed gain parameter alpha (α), that is generally user fixed or can be obtained on trial and error basis. Although several techniques are proposed in the literature but they suffer from certain issues, such as optimizing membership function in the fuzzy logic-based adaptation technique or being dependent on an external sensor [31].

Let us start by defining the orientation of the vehicle with respect to Inertial frame of reference. The angle along the longitudinal axis with horizontal plane is roll angle ∅ and the angle around the lateral axis with horizontal plane is called pitch θ. Considering the roll angles and road bank angles the following relations hold.

(31) $$\emptyset^{.}=\omega_{x}+\sin\emptyset {\tan\theta \omega }_{y}+\cos\emptyset {\tan\theta \omega }_{z}$$

(32) $$v_{y}{}^{.}=-\omega_{z}v_{x}+\omega_{x}v_{z}+a_{y}+\mathrm{gsin}\emptyset \cos\theta$$

(33) $$v_{x}^{.}=\omega_{z}v_{y}-\omega_{y}v_{z}+a_{x}+\mathrm{gsin}\theta$$

where $\omega_{x},\omega_{y},\omega_{z}$ are rotation rates along x, y, and z-axis correspondingly. The velocity components around center of mass are $v_{x},{\;v}_{y}$, and $v_{z}$. Further, $a_{x}$ and $a_{y}$ denotes acceleration components for center of mass inclusive of all the gravity effects.

Roll rate integration can be expressed using Equation (35).

(34) ∅int=∫0tωxc(τ)dτ

Simplifying the equations further, we assume that the pitch angle is very small so we can write tanθ≅θ. Let’s assume that the roll angle will have a moderate value cosθ=1 and vertical component $v_{z}$ is also small. Then, Equations (32)–(34) can be expressed as:

(35) $$\emptyset^{.}=\omega_{x}+{\theta \omega }_{z}$$

(36) $$a_{y}=v_{y}{}^{.}+\omega_{z}v_{x}-\mathrm{gsin}\emptyset$$

(37) $$a_{x}=v_{x}{}^{.}+\omega_{z}v_{y}-\mathrm{gsin}\theta$$

The term $\sin\emptyset {\tan\theta \omega }_{y}$ is neglected in Equation (31) since it is a small value of higher order.

Now the following observations would hold:

- The product of pitch angle and yaw rate is small. The roll angle can be obtained by integration of the roll rate obtained by the gyroscope.
- If the vehicle is in steady state condition, the time derivative of $v_{y}$ would be small and roll angle can be calculated through Equation (37).
- Pitch angle can be determined from the Equation (38) when roll angle gets known.

#### 2.3.2. Kalman Filter Implementation

The Kalman filter utilizes the state estimate of a discrete time system that can be written as a linear stochastic difference equation along with measurement equation as following:

(38) $$x_{k+1}=A_{k\;}x_{k}+{B\;u}_{k}+w_{k}$$

(39) $$z_{k}=H_{k\;}x_{k}+v_{k}$$

where x ∈R represents the system state, A is called system Matrix, B is said to be input matrix, H is the output matrix, U is the input to the system, $v_{k}$ is known as measurement noise, $w_{k}$ is called process noise, $z_{k}$ is the output of the system.

Further, $w_{k}$ and $v_{k}$ are the two random variables and are assumed to have Gaussian distribution with zero mean, they can be written as p(w)−N(0,Q); p(v)−N(0,R).

Real Time Implementation:

Real time implementation involves the following steps.

Step 1: ${\hat{\theta }}_{k|k-1}$ is priori angle estimate which will be equal to previous state estimate ${\hat{\theta }}_{k-1|k-1}$ with addition of unbiased rate times Δt. Since bias cannot be measured directly, the estimate of priori bias will be equal to the previous bias.

Prediction

Current State

(40) $${\hat{x}}_{k:k-1}=F{\hat{x}}_{k-1:k-1}+{B\theta }_{k}^{.}$$

(41) $${\left[\begin{array}{c} \theta \\ \theta_{b}^{.} \end{array}\right]}_{k\vdots k-1}=\left[\begin{array}{cc} 1 & -\Delta t\\ 0 & 1 \end{array}\right]{\left[\begin{array}{c} \theta \\ \theta_{b}^{.} \end{array}\right]}_{k-1\vdots k-1}+\left[\begin{array}{c} \Delta t\\ 0 \end{array}\right]\theta_{k}^{.}$$

Error Co-Variance

(42) $$P_{k\vdots k-1}={\mathrm{FP}}_{k-1|k-1}F^{T}+Q_{k}$$

Measurement Update

$$z_{k}-\left[1\;0\right]{\left[\begin{array}{c} \theta \\ \theta_{b}^{.} \end{array}\right]}_{k\vdots k-1}$$

$$z_{k}-\theta_{k|k-1}$$

Innovation Covariance

$$S_{k}={\mathrm{HP}}_{k|k-1}H^{T}+R$$

$$P_{00\;k|k-1}+R$$

(43) $$P_{00\;k|k-1}+\mathrm{var}\left(v\right)$$

Calculating Kalman Gain

(44) $$K_{k}=P_{k|k-1}H^{T}S_{k}^{-1}$$

Posteriori Estimate

(45) $${\hat{x}}_{k|k}={\hat{x}}_{k|k-1}+K_{k}y_{k}^{\sim }$$

(46) $${\left[\begin{array}{c} \theta \\ \theta_{b}^{.} \end{array}\right]}_{k\vdots k}={\left[\begin{array}{c} \theta \\ \theta_{b}^{.} \end{array}\right]}_{k\vdots k-1}+\left[\begin{array}{c} K_{0}\\ K_{1} \end{array}\right]y_{k}^{\sim }$$

Update

(47) $$P_{k|k}=\left(I-K_{k}H\right)P_{k|k-1}$$

## 3. Experiment and Results

### 3.1. Model Validation

CarSim^®^ (CarSim, Ann Arbor, MI, USA) is a system-level vehicle dynamics simulation software that is widely used in automotive industry. CarSim^®^ is the “company standard” vehicle dynamics software in several automotive companies, and is in use for various purposes at many more of the world’s OEM companies and their suppliers. The same has been used in [32,33] to simulate the results. We have developed the vehicle’s analytical model that was simulated in MATLAB^®^ (The MathWorks, Inc., Torrance, CA, USA). Validation is performed by conducting several standard tests and comparing them with CarSim^®^ including step steer, Fishhook, and Double lane change.

### 3.2. Simulated Results for Luenberger & Sliding Mode Observer

**Figure 8:**
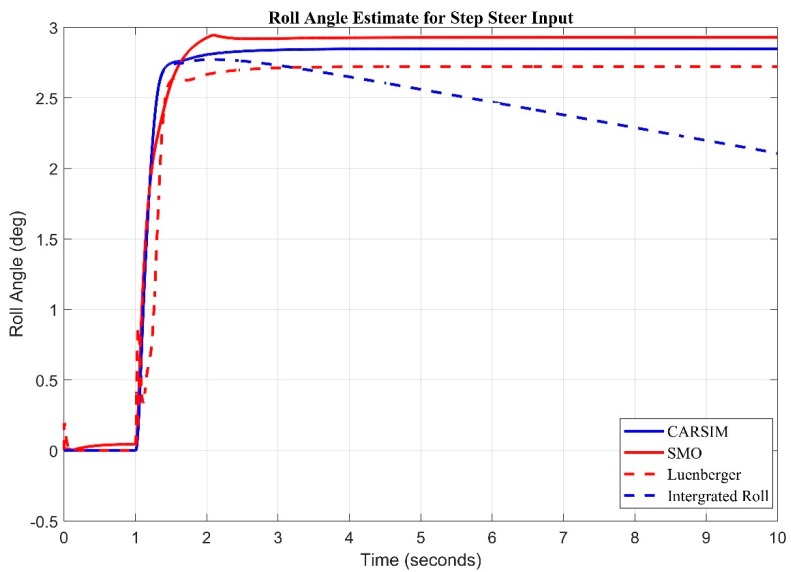
Observer results for step input estimate for roll angle.

ISO (International Organization for Standardization) Step Input is International Standard that specifies open-loop test methods for determining the transient response behavior of road vehicles. It is applicable to passenger cars, as defined in ISO 3833, and to light trucks. A step steer input of 100-degree step is fed to both observers and Carsim^®^ against which a roll angle of around 3 degree is observed as seen in Figure 8. It is observed that the sliding mode observer performed better by keeping better track of roll angle as compared to Luenberger during step steer input. The root mean square error obtained for sliding mode observer is 0.0906 degree where as Luenberger came up with a root mean square error of 0.2127 degrees.

**Figure 9:**
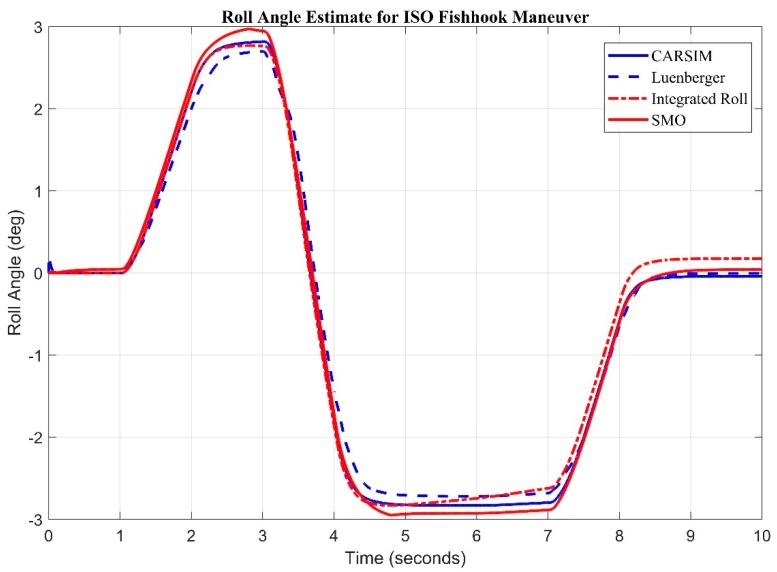
Observer Results for ISO Fishhook Input estimate for Roll Angle.

NHTSA (National Highway Traffic Safety Administration) released details of a dynamic maneuver designed to determine a vehicles susceptibility to rollover. The maneuver is known as the Fishhook (nhtsa.gov). Considering the NHTSA Fishhook maneuver (Figure 9) both sliding mode and Luenberger are fed with roll rate and lateral acceleration as input. It is again witnessed that the sliding mode observer is keeping better track of roll angle and root mean square error between sliding mode observer and Luenberger observer as compared to CarSim^®^ is found to be 0.0872 degrees and 0.1458 degrees respectively.

**Figure 10:**
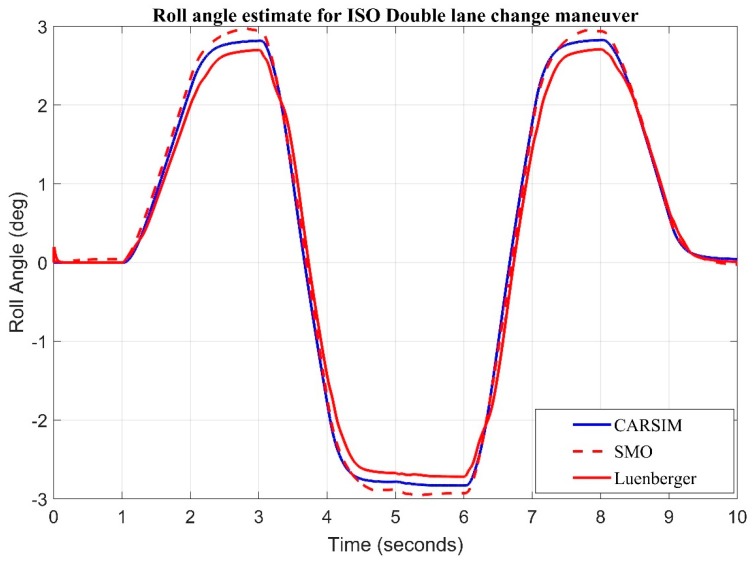
Observer results for ISO double lane change input estimate of roll angle.

ISO Double lane change specifies the dimensions of the test track for a closed-loop test method to subjectively determine a double lane-change which is one part of the vehicle dynamics and road-holding ability of passenger cars. It is applicable to passenger cars. ISO Double Lane change input (Figure 10) for roll rate and acceleration is fed to sliding mode and Luenberger observer. Root mean square error for Sliding mode observer is 0.0898 degree while the root mean square error for Luenberger observer is 0.1823 degree. This clearly indicates that the sliding mode observer tracks the roll angle better in comparison to Luenberger.

**Figure 11:**
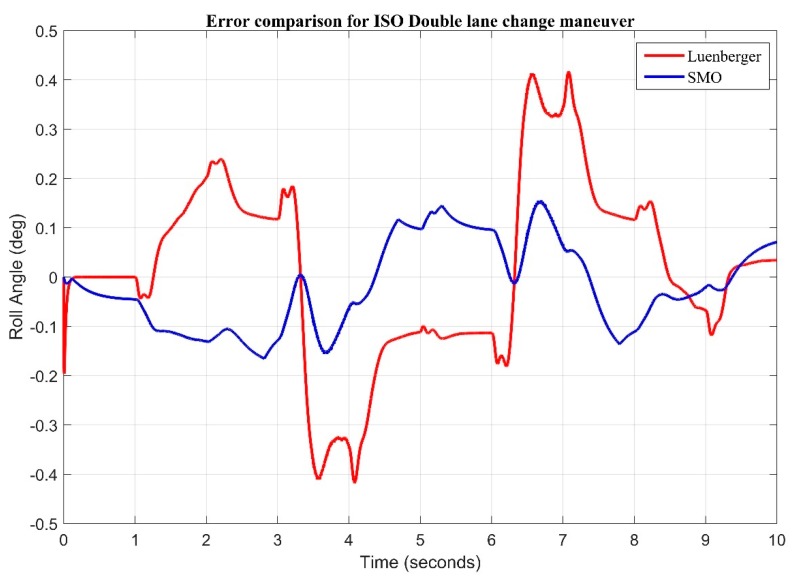
Error comparison of observers during double lane change input.

The error comparison plot for ISO double lane change maneuver is shown below in Figure 11.

**Figure 12:**
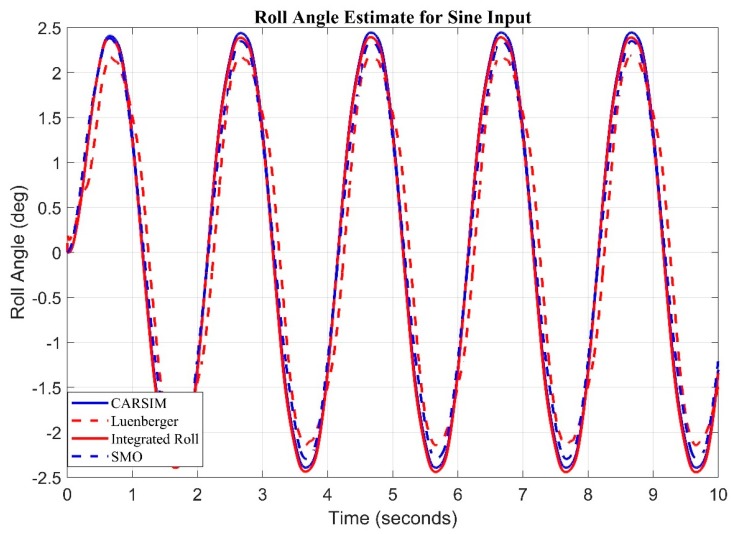
Observer Results for sine input estimate of Roll Angle.

Finally, altered Sine with dwell stability control testing (ISO 19365:16) for roll rate and acceleration is fed to observers, as seen in Figure 12. Sliding mode observer tracked the roll angle with Root mean square error of 0.1537 and Luenberger produced root mean square error of 0.4471.

Table 2 shows the root mean square error (RMSE) comparison of Luenberger and sliding mode observer. We can see that the RMSE is less in SMO as compared to Luenberger Observer.

**Figure 13:**
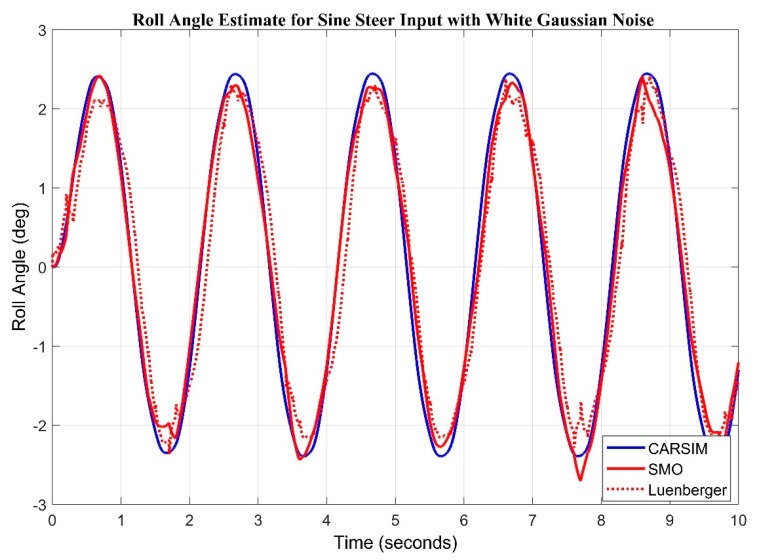
Observer Results for Sine input with Gaussian noise.

**Figure 14:**
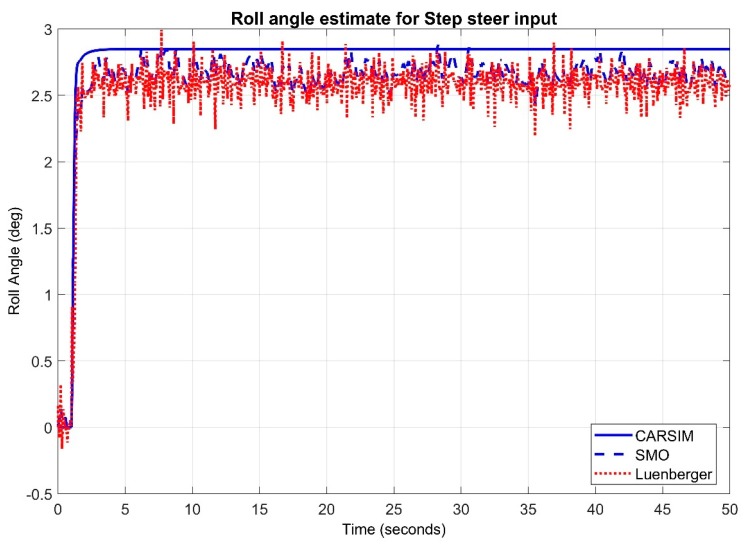
Observer Results for Step input with Gaussian noise.

Max error in case of step input remained 0.2223 for sliding mode observer whereas, maximum error in case of step input for Luenberger is 0.3126. We investigated the response of observers in presence of white Gaussian noise. Noise of magnitude 0.01 is introduced along the sine wave input in Figure 13 and step input in Figure 14. It can be clearly observed that sliding mode observer keeps better track of the system as compared to Luenberger observer.

Simulation results prove that Luenberger observer (LO) and sliding mode observer (SMO) exhibit strong robustness to variations. SMO may have better performance compared to LO because of the inherent properties of sliding mode theory.

### 3.3. Simulated Results Comparison of Complementary Filter with CARSIM^®^

**Figure 15:**
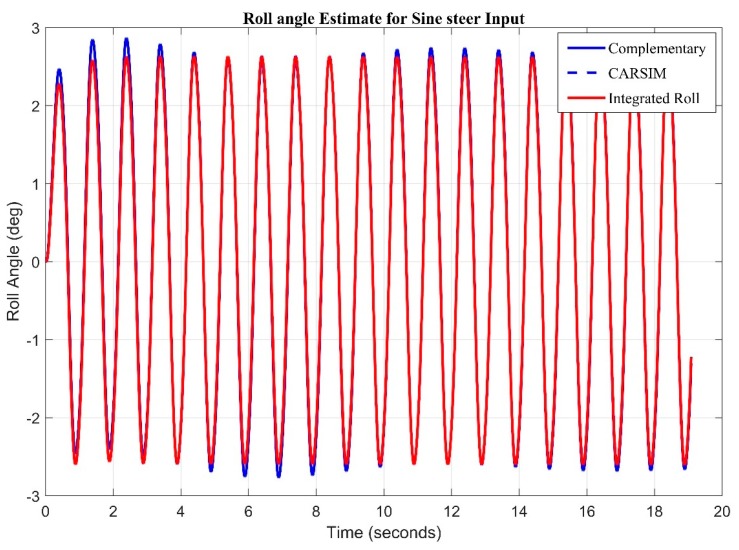
Complementary Filter Response for Sine Input.

A swept sine having 0.5 Hz frequency and amplitude of 100 deg is fed as a steer input (Figure 15). It can be seen from figure that the roll angle obtained from pure integration behaves in similar manner to the roll angle obtained from blend of two equations using high pass and low pass filters. This is because, during fast maneuvers, there is less drift produced in gyroscope measurements.

**Figure 16:**
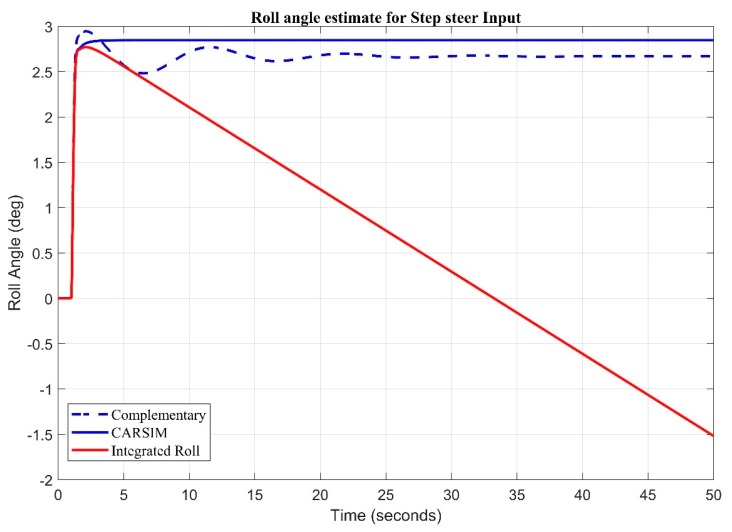
Complementary Filter Response for Step Steer Input.

A 100-degree step steer input is fed to system and it is clearly seen that during the step maneuver the angle obtained from the pure integration of roll rate through the gyroscope suffers from drift, whereas the blend recovers within a short time to keep track of the roll angle (Figure 16). 

### 3.4. Real Time Complemenary Filter Implementation

#### Experimental Setup

**Figure 17:**
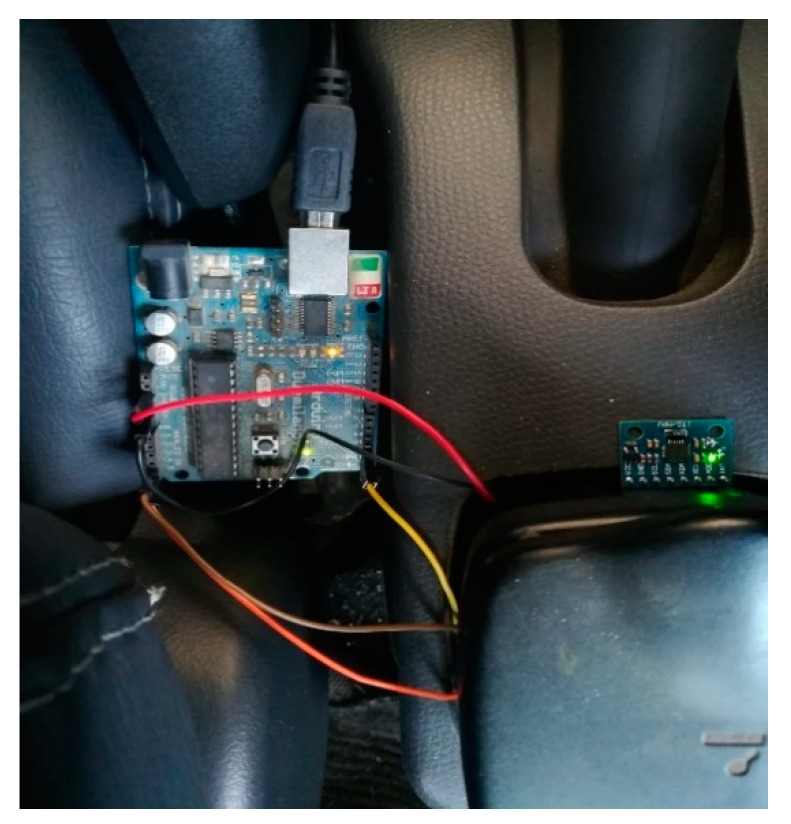
Experimental setup containing Arduino and MPU6050.

The experimental setup containing arduino duemilanove and MPU6050 can be seen in Figure 17. The sensor is mounted at approximate Center of gravity location of vehicle. Arduino Duemilanove is a single-board computer manufactured by Arduino in Italy. It can be powered by either a USB connection or with an external power supply. The detailed description and specifications can be seen in appendix. The MPU-6050 sensor contains a Digital Motion Processor™ (DMP™), which processes complex six-axis Motion Fusion algorithms. The inner DMP uses a high-performance algorithm to fuse accelerometers measure data and gyroscope measure data and output attitude quaternion which has a good real-time performance.

**Figure 18:**
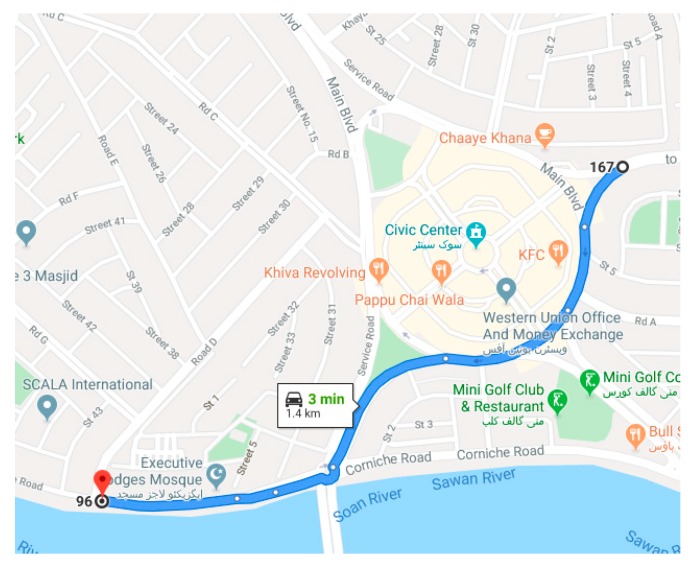
Test Track Map (Zoom Scale 500 ft).

**Figure 19:**
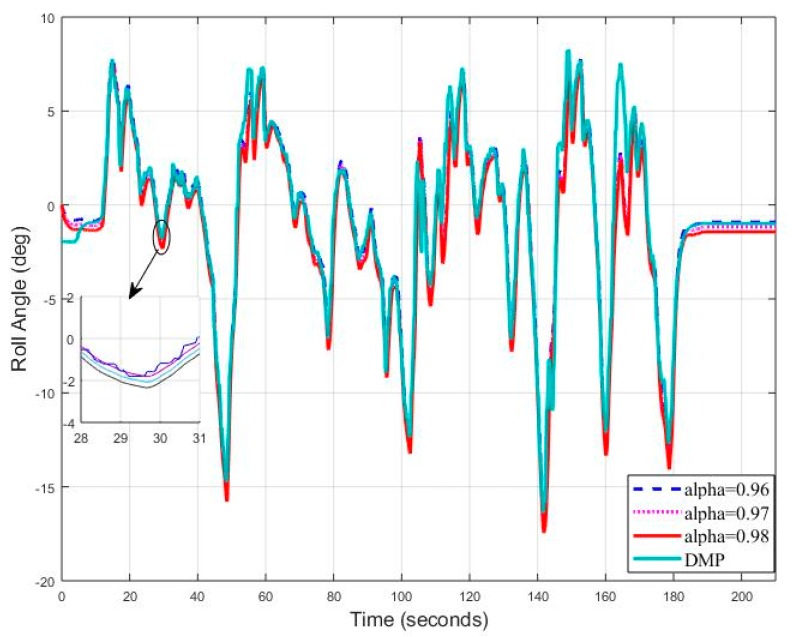
Real Time Roll Angle Estimate at f = 5 Hz for Complementary Filter.

Figure 18 shows the test track details obtained by google maps. Let’s now consider the starting frequency of 5 Hz and observe the roll angle behavior using different tuning parameters of filter. A slight deviation can be seen in Figure 19 for (DMP™) data as it takes 10–15 s to settle down [34]. We observed the root mean square error for roll angle estimate to be 1.1693 degrees.

**Figure 20:**
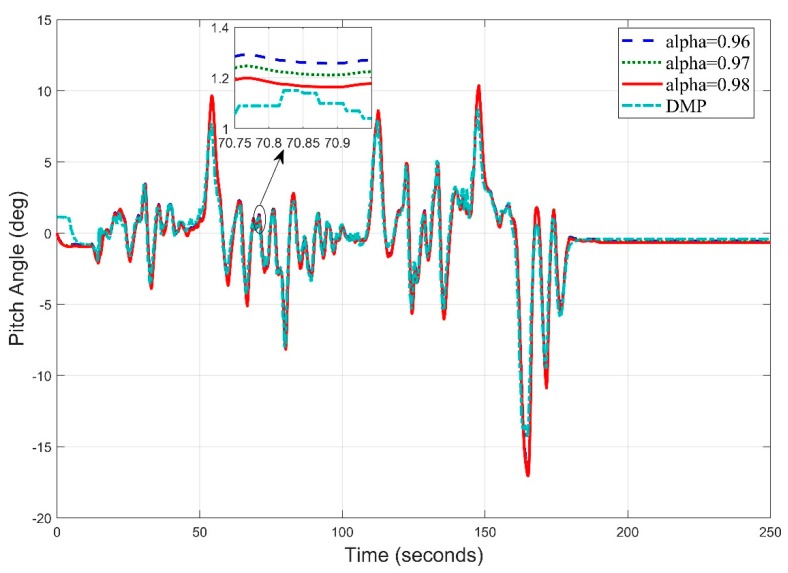
Real Time Pitch Angle Estimate at f = 5 Hz for Complementary Filter.

Similarly, in Figure 20. We obtain the pitch angle response by setting frequency at 5 Hz. The Digital Motion Processor (DMP) takes 10–15 s to stabilize. This time is utilized by the algorithm to calculate gyro and accelerometer offsets to remove zero error.

The pitch angle estimation appears precise at most of the points with an RMS error value of 0.8951.

**Figure 21:**
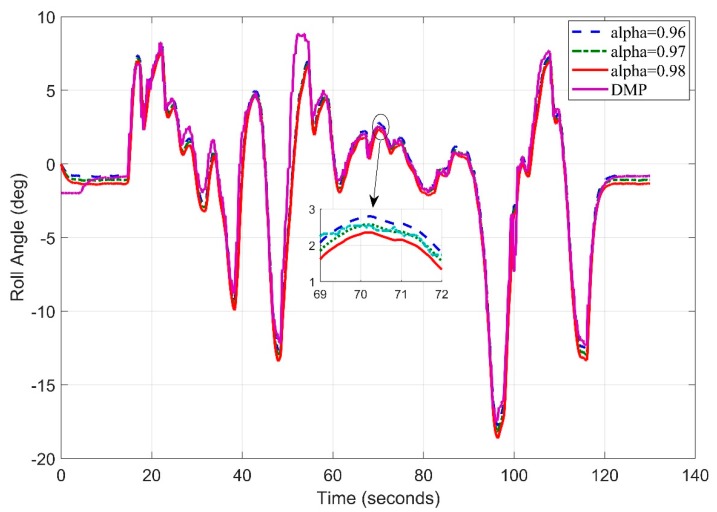
Real Time Roll Angle Estimate at f = 10 Hz for Complementary Filter.

Repeating the procedure again, this time frequency is set to 10 Hz. We found that the roll angle error has decreased and now root mean square error is 1.0944 degrees (Figure 21).

**Figure 22:**
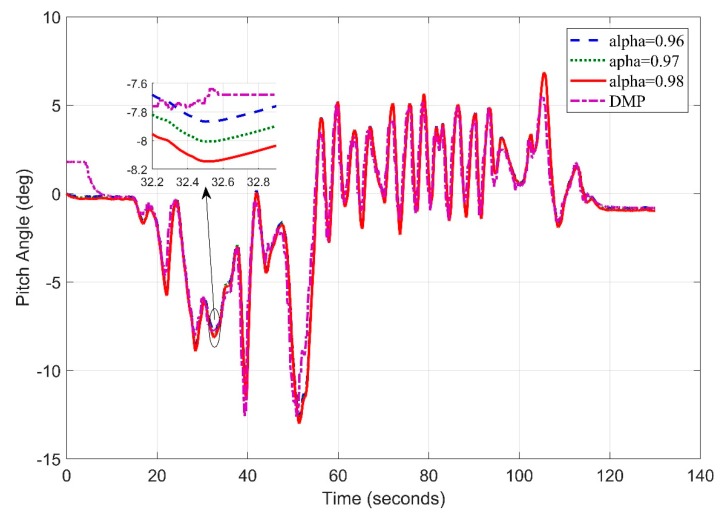
Time Pitch Angle Estimate at f = 10 Hz for Complementary Filter.

Similarly, the pitch angle estimate (Figure 22) obtained at f = 10 Hz has the root mean square error of 0.9969 degree. This means, at this frequency, the roll angle result has improved from the roll angle result at 5 Hz frequency.

**Figure 23:**
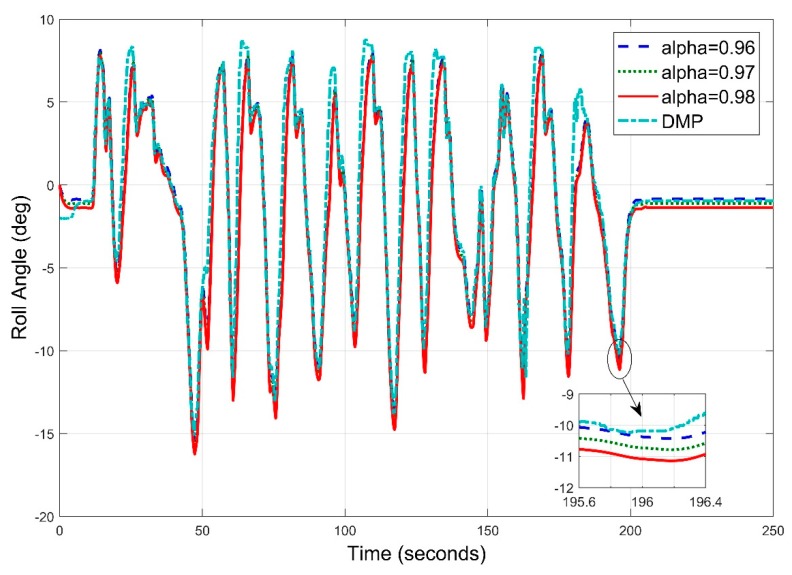
Real time roll angle estimate at f = 20 Hz for complementary filter.

Increasing the set frequency to 20 Hz (Figure 23), we concluded that the root mean square error between digital motion processor and the best value of alpha 0.96 increased to 1.8868 degrees, which is already greater than the roll angle errors obtained under previous frequencies.

**Figure 24:**
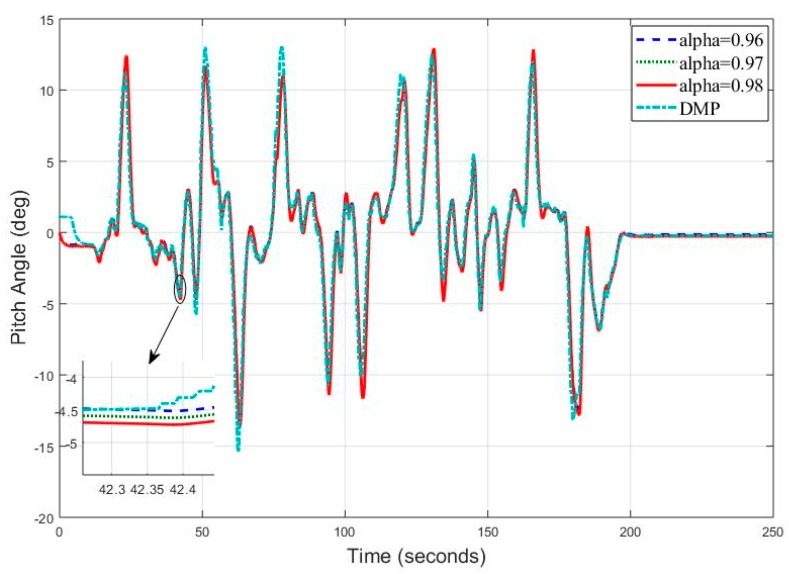
Real Time Pitch Angle Estimate at f = 20 Hz for Complementary Filter.

Setting up the frequency at 20 Hz (Figure 24), we estimated the pitch angle using different filter tuning parameters and found that the root mean square is now 1.1371 degrees, which is greater than the previous two readings, i.e., frequency of 5 Hz and 10 Hz.

**Figure 25:**
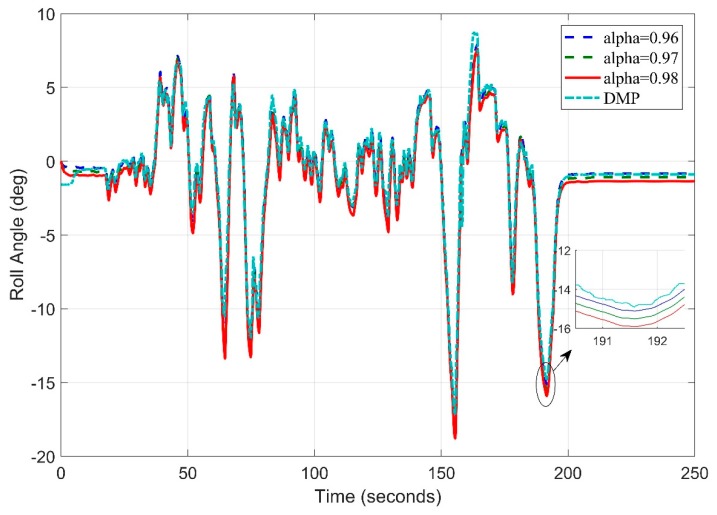
Real Time Roll Angle Estimate at f = 25 Hz for Complementary Filter.

Repeating the experiment again, we now set the sampling frequency to 25 Hz and estimated roll angle at different values of alpha with new sample frequency. It has been observed that the roll angle results (Figure 25) are much better than previous frequency results in terms of root mean square error. Looking at the graph, we can interpret that system kept track of angle in almost all the situations. We found that the root mean square has decreased to 0.6738 degrees for roll angle estimates.

**Figure 26:**
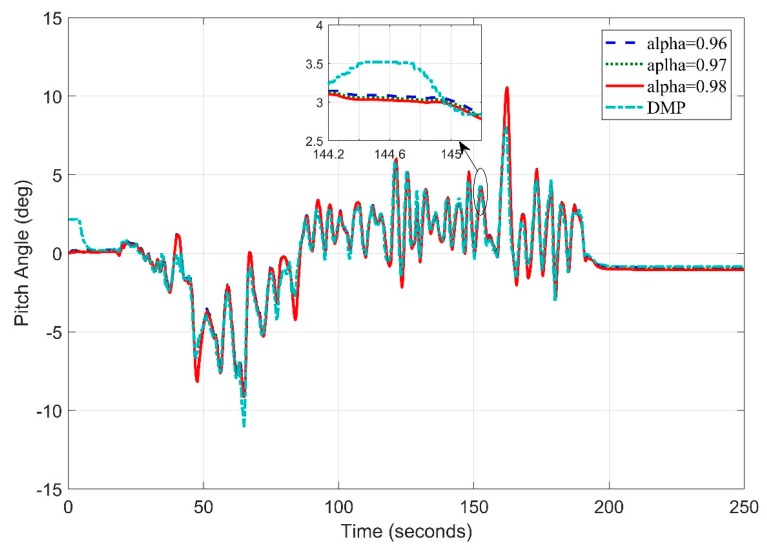
Real Time Pitch Angle Estimate at f = 25 Hz for Complementary Filter.

Similarly, we analyzed the estimation results for pitch angle at frequency 25 Hz, we can clearly see in Figure 26 that complementary filter tracked roll angle at almost all data points. Observed root mean square error is now 0.7280 degrees for roll angle, which is also smaller less than all the frequency data collected before. We can see a slight deviation of digital motion processor data in the start since it takes 10–15 s to settle down the values to true values.

**Figure 27:**
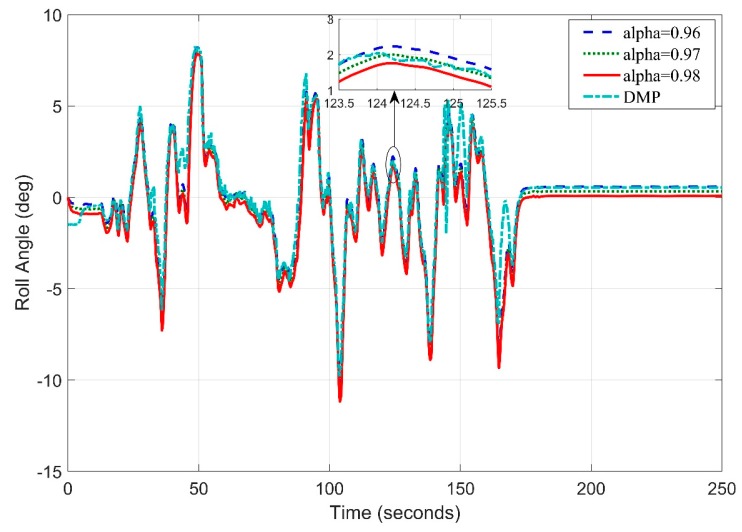
Real Time Roll Angle Estimate at f = 40 Hz for Complementary Filter.

Frequency is now set to 40 Hz for roll angle estimate using different filter tuning parameters (Figure 27). Roll angle root mean square error is now 1.0140, which is greater than the results obtained at frequency 25 Hz.

**Figure 28:**
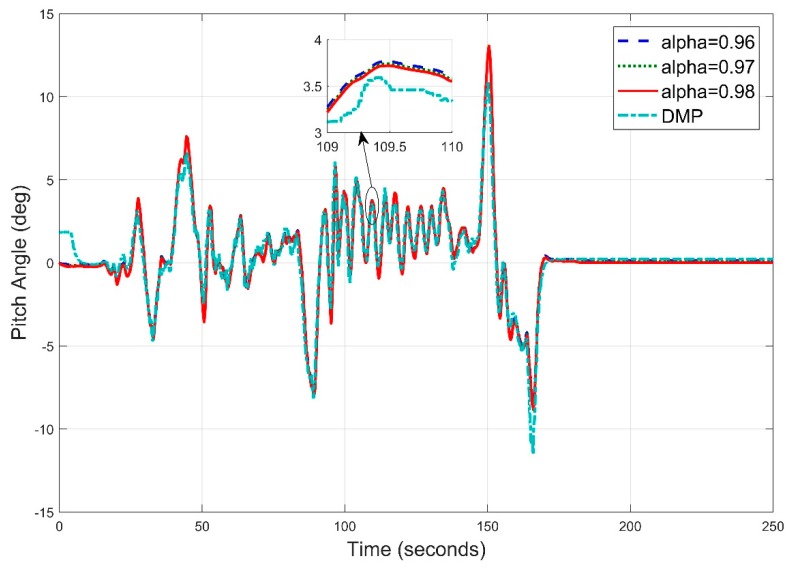
Real Time Pitch Angle Estimate at f = 40 Hz for Complementary Filter.

We set the sampling frequency to 40 Hz now. The RMSE now is smaller for pitch angle estimate (Figure 28) but still results are not better than 25 Hz frequency. The RMSE now obtained is 0.7657 degrees for pitch angle estimation.

### 3.5. Real Time Complemenary Filter Implementation

A Kalman filter with the following parameters is applied on a digital controller and InvenSense MPU-6050 (TDK InvenSense) is mounted on the vehicle. The sampling frequency is 25 Hz. The tuning parameters used are as follows: Q_angle = 0.0005; Q_bias = 0.0015; R_measure = 0.015.

The Allan variance (AV) is a well-known technique that is commonly used to identify and to quantify inertial sensors’ stochastic noises, as quantization noise, random walks errors, and bias instability, among others. It is mandatory to process data from static measurements in order to apply Allan variance [35]. Theoretical details about the AV technique can be found in the literature [36,37] and are beyond the scope of this paper.

**Figure 29:**
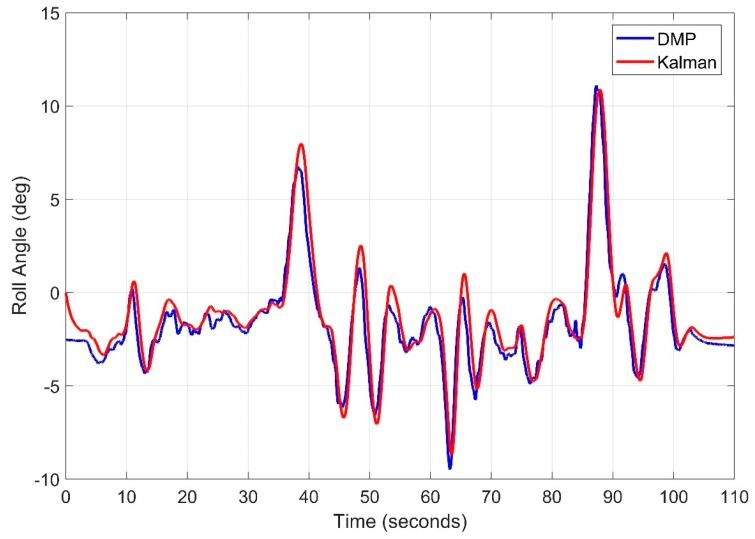
Real Time Roll Angle Estimate for Kalman Filter.

**Figure 30:**
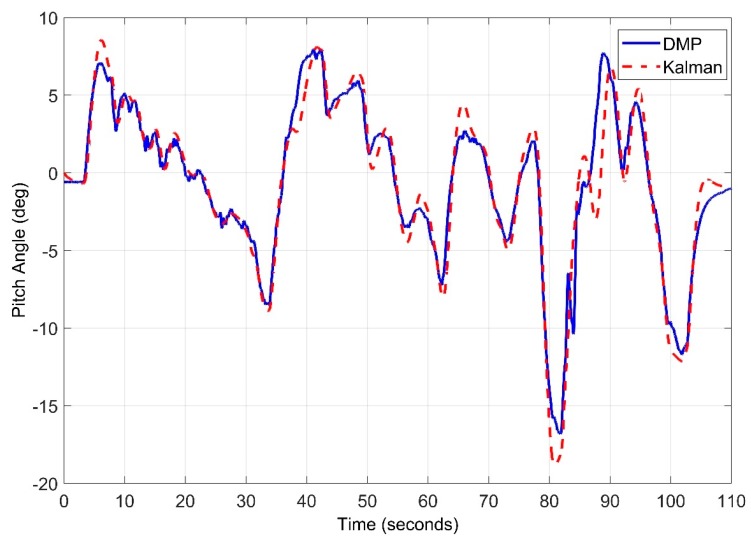
Real Time Pitch Angle Estimate for Kalman Filter.

While observing the roll angle a root mean square of 0.9026 degrees is obtained. As seen in Figure 29. In contrast, the pitch angle root mean square error is 0.9213 degrees as seen in Figure 30. Kalman filter can be further tuned to get more precise results, however, algorithm complexity and computation burden is involved for real time implementation.

**Figure 31:**
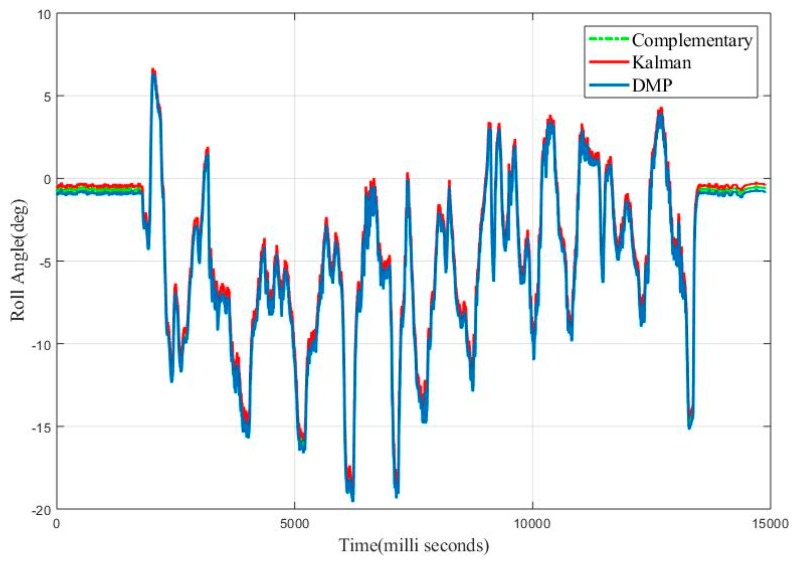
Real Time Roll Angle Estimate for Complementary and Kalman Filter.

To investigate the efficacy of the complementary filter, we applied Kalman and complementary filter simultaneously to evaluate the vehicle roll angle. Figure 31 shows the results obtained for vehicle roll angle. The RMSE obtained for the complementary filter is 0.61 degrees and for Kalman filter is 0.70 degree.

**Figure 32:**
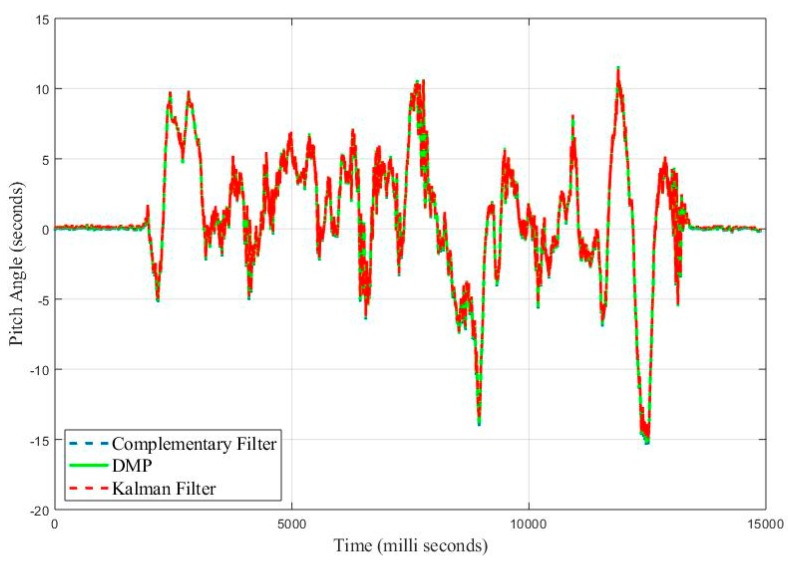
Real Time Pitch Angle Estimate for Complementary and Kalman Filter.

Figure 32 shows the response of both Kalman and Complementary filter with respect to DMP data when pitch angle is estimated. RMSE obtained for Kalman filter is 0.69 degree, whereas RMSE for complementary is 0.596 degrees.

## 4. Discussion

Attitude estimation in ground vehicle is challenging due to rough terrain and noise. Table 3 below shows RMSE comparison of our research with current literature. Algorithms used in these are mainly genetic algorithm (GA), Bayesian propagation (BP), neural networks (NN), unscented Kalman filter (UKF), extended kalman filter (EKF), non-linear complementary filter (NCF), and differential nonlinear complementary filter.

The results demonstrated by [38] proposed that attitude prediction can be completed 2 s ahead of the vehicle motion. The mean relative error (MRE) of the predicted roll angle is 0.0383 and predicted pitch angle is 0.0476 with. RMSEs of 1.8° for roll and 2.1° for pitch angle. The computational cost with higher RMSE is the disadvantage of the discussed approach. Research proposed by [24] used Kalman filtering with GPS as an additional sensor adding increased computation cost. The efficacy of algorithm was tested on straight track. In the research carried out by [39], GPS was used as an additional sensor along with IMU. Kalman filtering was applied to fuse data together. GNSS along with IMU was used by [40], but initially, the system takes 200 s to stabilize the values. A pseudo algorithm was developed by [41] which is an extension of Whaba’s problem. The system utilized quaternion and the algorithm was tested using a 3.2 Ghz core I7, decreasing the computation time. The RMSE using pseudo approach was 1.3 degree for roll angle and 1.1 degree for pitch angle. A nonlinear complementary filter and differential nonlinear complementary filter were used by [42] to estimate roll and pitch for UAV. The fusion algorithm took 41 ms of computational time which is still large as compared to the techniques mentioned in literature. EKinox D IMU along with MPU 6000 was used by [43] for comparison purpose. The Roll angle RMSE obtained using MPU6000 and U-Blox GNSS was 1.6 degree whereas for pitch angle RMSE was found 1.8 degree. Low RMSE mentioned in table against this research is the result from EKinox, such high-end equipment cannot be used in commercial vehicles as the sensor costs 47,000 USD.

Our proposed filter is fast since it is free of iteration. To decrease the significant effects of bias imposing on gyroscope, bias compensation is merged with filtering architecture. Several experiments are carried out on real hardware in a real time environment to show the performance comparison. Results show that the proposed Filters can reach the accuracy of Kalman filter and higher order observers. Successfully tested in ordinary road conditions we find a balance between estimation accuracy and time consumption. Compared with iterative methods, the proposed filter has much less convergence time.

The results clearly indicate that, during real time implementation of second order complementary filter, the root mean square error of 0.62 degrees is observed for roll angle estimate. The implementation of a first order complementary filter is easier, simpler, and works well during static maneuvers, however, in the presence of road disturbances, the performance is deteriorated. If we decrease the value of the filter tuning parameter, it adds more weight to the static equation, and the filter will become non-responsive during very fast maneuvers. So, we set the parameters according to system requirements so that balance between static and dynamic motion results is maintained and estimation accuracy is not affected. The optimal sampling frequency obtained for Complementary filter through the experimentation in real time environment with sensor mounted near center of gravity of vehicle is 25 Hz as it brings small root mean square error while on all other frequency errors are larger. The results obtained demonstrate that the complementary filter is equally good as the Kalman filter with less computation burden and better accuracy, with the least tuning involved. Finally, the implementation of the proposed algorithms can be easily carried out on low cost hardware that can be easily mounted on standard commercial vehicle with the addition of marginal cost. Further, based on attitude information, anti roll systems can be designed and implemented.

## 5. Conclusions

In the research carried out, estimation of vehicle roll angle is investigated with the help of various schemes available in the literature. Real time implementation is also carried out using multi-axis gyroscope and accelerometers. The estimation of roll angle done using this approach is used to remove the gravity component from lateral acceleration prior to estimation of vehicle’s lateral velocity. The given estimation method is simple and cost effective as it does not require any tire models or complex vehicle models. The major advantage of the proposed technique is fast and precise estimation up to error of 0.7 degrees by adding marginal cost and equipment with the least computational complexity involved. The algorithm is tested in ordinary driving conditions with speeds varying from 0 to 90 Km/hr. The estimate of roll and pitch angles, along with lateral acceleration, in a real time environment can aid the estimation of other vehicle and tire road interaction phenomena. This simple technique can be coupled with a sliding mode observer to support the highly precise estimation of vehicle dynamics. Finally, it can be safely claimed that in the presence of ordinary road conditions, this technique can easily provide a precise estimate of the roll angle by adding marginal cost to the user.

## Figures and Tables

**Table 1:** Specifications comparison of different Gyroscopes.

Sno.	Name	Manufacturer	Technology	$\mathrm{Noise}\;\mathrm{Density}\;dps/\sqrt{Hz}$
1	TG6000	KVH (Middle Town, CT, USA)	Fiber Optic	0.001
2	HG1700AG37	Honeywell (Charlotte, NC, USA)	Ring Laser	0.002
3	VG700MB	Cross Bow (San Jose, CA, USA)	Fiber Optic	0.006
4	HG1700AG68	Honeywell (Charlotte, NC, USA)	Ring Laser	0.008
5	LandMark10	Gladiator Tech (Snoqualme, WA, USA)	MEMS	0.012
6	ADIS16355	Analog Devices (Norwood, MA, USA)	MEMS	0.033
7	MTi-1	Xsens (Enschede, The Netherlands)	MEMS	0.01
8	L3GD20	ST Microelectronics (Geneva, Switzerland)	MEMS	0.03
9	MPU-6050	TDK-InvenSense (San Jose, CA, USA)	MEMS	0.005

**Table 2:** RMSE comparison for Luenberger and Sliding mode observer in deg.

Input	Rms Error Deg (SMO)	Rms Error Deg (Luenberger)	Maximum Error (SMO)	Maximum Error (Luenberger)
Step Input	0.0906	0.2127	0.2223	0.3126
Sinusoidal Input	0.1537	0.4471	0.2936	0.6742
ISO Fish Hook Maneuver	0.0872	0.1458	0.1415	0.2132
ISO Double Lane Change	0.0898	0.1823	0.2074	0.2487

**Table 3:** RMSE comparison with current literature.

Author	Estimation Parameter	Platform	Estimator	Computation Cost	Error Max (RMSE) (deg)
Qingyuan Zhu et al. [38]	Roll	Prototype Vehicle	GA	100 ms	1.8 (Roll)
	Pitch		BP NN		2.1 (Pitch)
Hamad Ahmed et al. [24]	Roll	Standard Vehicle	KF	20–25 ms	0.1 (Roll)
	Pitch				0.13 (Pitch)
	Yaw				0.01 (Yaw)
Javier Garcia Guzman et al. [39]	Roll	Standard Vehicle	KF	14.2 ms	0.76 (Roll)
	Pitch		UKF	6.76 ms	0.63 (Pitch)
Daehee Won et al. [40]	Roll	Standard Vehicle	EKF	21.4 ms	0.28 (Roll)
	Pitch				0.55 (Pitch)
RobertoG.Valenti et al. [41]	Roll	Standard Vehicle	Pseudo	1.42 μs	1.32 (Roll)
	Pitch		Madwick		1.19 (Pitch)
	Yaw		EKF		
XudongWen et al. [42]	Roll	UAV	NCF	41 ms	1.16 (Roll)
	Pitch		DNCF		0.50 (Pitch)
	Yaw		-		-
Rodrigo Gonzalez et al. [43]	Roll	Standard Vehicle	KF	0.2 s	0.362 (Roll)
	Pitch				0.339 (Pitch)
	Yaw				1.839 (Yaw)
Proposed scheme	Roll	Standard Vehicle	CF	3.2 ms	0.6738 (Roll)
	Pitch				0.7280 (Pitch)
